# Differences in Trunk Kinematic between Frail and Nonfrail Elderly Persons during Turn Transition Based on a Smartphone Inertial Sensor

**DOI:** 10.1155/2013/279197

**Published:** 2013-11-28

**Authors:** Alejandro Galán-Mercant, Antonio I. Cuesta-Vargas

**Affiliations:** ^1^Department of Physical Therapy, School of Medicine, University of Málaga, 29071 Málaga, Spain; ^2^School of Clinical Sciences of the Faculty of Health at the Queensland University of Technology, Brisbane, QLD 4000, Australia

## Abstract

*Objective*. Firstly, to, through instrumentation with the *iPhone4* smartphone, measure and describe variability of tridimensional acceleration, angular velocity, and displacement of the trunk in the turn transition during the ten-meter Extended Timed-Get-up-and-Go test in two groups of frail and physically active elderly persons. Secondly, to analyse the differences and performance of the variance between the study groups during turn transition (frail and healthy). *Design*. This is a cross-sectional study of 30 subjects over 65 years, 14 frail subjects, and 16 healthy subjects. *Results*. Significant differences were found between the groups of elderly persons in the accelerometry (*P* < 0.01) and angular displacement variables (*P* < 0.05), obtained in the kinematic readings of the trunk during the turning transitions. The results obtained in this study show a series of deficits in the frail elderly population group. *Conclusions*. The inertial sensor found in the *iPhone4* is able to study and analyse the kinematics of the turning transitions in frail and physically active elderly persons. The accelerometry values for the frail elderly are lower than the physically active elderly, whilst variability in the readings for the frail elderly is also lower than the control group.

## 1. Background

Clinical frailty syndrome is a common geriatric syndrome which is characterized by physiological reserve decreases and increased vulnerability and which may, in the event of unexpected intercurrent processes, result in falls, hospitalization, institutionalization, or even death [1]. The changes associated with ageing and frailty are associated with changes in gait characteristics and the basic functional capacities of the individual [2]. This variability in different movement patterns has been interpreted as a more conservative gait pattern in order to increase gait stability and reduce the risk of falls [3]. This new, more conservative gait pattern has greater cognitive involvement and produces a result focused entirely on movement, whilst the perception of unexpected trigger factors may be overlooked [4]. Dual tasks have been shown to affect normal gait development even in healthy persons [5].

Turning while walking is a common occurrence in everyday life [6]. Turning requires transfer and rotation of the body towards the new walking direction while maintaining dynamic stability [7]. The Timed Get Up and Go (TGUG) test is a widely used tool to evaluate balance and some functional tasks through clinical evaluation of mobility and the risk of falls [2, 8–10]. The clinical potential of the TGUG test comes from the possibility of sequencing several basic functional abilities, such as standing up and sitting down transitions, and transitions which require balance, such as turning [11]. The TGUG test, despite being widely used in clinical practice, has limitations. As a consequence, the TGUG test is currently carried out in an instrumented manner by attaching inertial sensors to the body [2, 9, 12–16].

The latest generation of smartphones often includes inertial sensors with subunits such as accelerometers and gyroscopes which can detect acceleration and inclination [17]. The apps developed for these smartphones mean the data from the accelerometer and the gyroscope these can be read, stored, transferred and displayed [18, 19]. These apps evaluate and assess kinematic variables related to gait [20], measures in the Cobb angles in X-rays, or provide an objective method to classify levels of physical activity and as indicator of the degree of functional capacity and quality of life [17, 21].

The goals of the present study are as follows. Firstly, to, through instrumentation with the *iPhone4* smartphone, measure and describe variability of tridimensional accelerations, angular velocity, and displacement of the trunk in the turn transition during the ten-meter Extended Timed-Get-up-and-Go test in two groups of frail and physically active elderly persons. Secondly, to analyse the differences and performance of the variance between the study groups during turn transition (frail and healthy).

## 2. Methods

### 2.1. Design and Participants

A cross-sectional study that involved 30 subjects over 65 years and 14 frail and 16 healthy elderly persons. The participants were classified with frailty syndrome by the Fried criteria (unintentional weight loss, self-reported exhaustion, weakness, slow walking speed, and low level of physical activity) [1]. Exclusion criteria werehistory of pain in the last twelve months, previous surgery, presence of a tumour, and musculoskeletal disorders in the upper or lower extremity. Patients with impaired cognition, musculoskeletal back comorbidities, and problems associated with exercise intolerance were also excluded. All participants were clinically examined by a physiotherapist, and none of them were found to have any exclusion criteria.  Table 1 shows the characteristics of the sample and stopwatch values in the ETGUG test.

Healthy elderly participants were recruited through notices at the Sport and Health Centre in Torremolinos, Spain. Frail elderly participants were recruited through notices at Geriatric Centres in Torremolinos and Benalmadena, Spain. Written informed consent was obtained from each individual. The study was approved by the Ethics Committee of the Faculty of Medicine at the University of Malaga, Spain.

### 2.2. Data Collection and Procedures

Linear acceleration was measured along three orthogonal axes using the *iPhone4* accelerometer snugly secured to the test subjects by a neoprene fixation belt over the sternum. Previous studies show that the essential spatiotemporal characteristics of overground walking can be obtained by trunk accelerometry; individual step or stride cycles can be identified, and fair estimations of step length and walking speed can be obtained using a single triaxial accelerometer [22].

The orientation and movement of the sensors are presented as roll, pitch, and yaw Euler angles (RPY). If the sensor's RPY axes are aligned with the anatomical axes of the trunk, the roll angle of a movement is around the anteroposterior (AP) axis, the pitch angle is around the left-right axis, and the yaw angle is around the vertical (V) axis.

This smartphone is equipped, as is the IC3, with three triaxial elements for the detection of kinematic variables: a gyroscope, a magnetometer, and an accelerometer. Apple uses an LIS302DL accelerometer in the *iPhone4* [23]. The application used to obtain kinematic data was* xSensor Pro, Crossbow Technology, Inc.*, available at the Apple *AppStore*. The *iPhone4* has storage capacity of 20 MB, and the data for each trial was transmitted as email for analysis and postprocessing. The data-sampling rate was set to 32 Hz. An *iPhone4* is required in order to obtain accelerometer, gyroscope, and magnetometer data together; earlier versions do not allow this possibility. A previous study showed an interobserver error (standard deviation of the difference between measurements by two different observers) of 4.0° for the iPhone and 3.4° for the protractor [17].

### 2.3. Extended Timed-Get-Up-and-Go Test

All subjects performed the Extended Timed-Get-up-and-Go test (ETGUG) three times, and the best trial was selected based on the total score for the full test. Devices were not removed between trials. Subjects had five minutes of rest between trials. All subjects used an armless chair and were instructed not to use their arms to stand up. Although in traditional ETGUG an armchair is used [24], we used an armless chair. The beginning and end of the walkway were marked with 2.5 cm green tape on the floor. The tape markings were shown to the subjects before the trials. Subjects were instructed to sit straight with their backs touching the back of the chair. Once the go signal was given by the tester, they stood up from the chair, walked as fast as possible but without running, turned left or right after passing the green tape at the end of the walkway, then returned to the chair, turned around, and sat down. The tester timed the performance with a stopwatch.

### 2.4. Turning Transitions of the Extended Timed-Get-Up-and-Go Test

The most important problem in analyzing turns is identifying the onset and offset of the turns. Offline data processing was used to identify the turning transition of the ETGUG test. The turn transition used in the study was the first one, the transition between the gait—go from the chair and the gait—come to the chair. The turning transition of the ETGUG test was detected with gyroscope data of the *iPhone4* accelerometer and was detected and analysed using a separate method [9].

### 2.5. Data Processing

**Figure 1:**
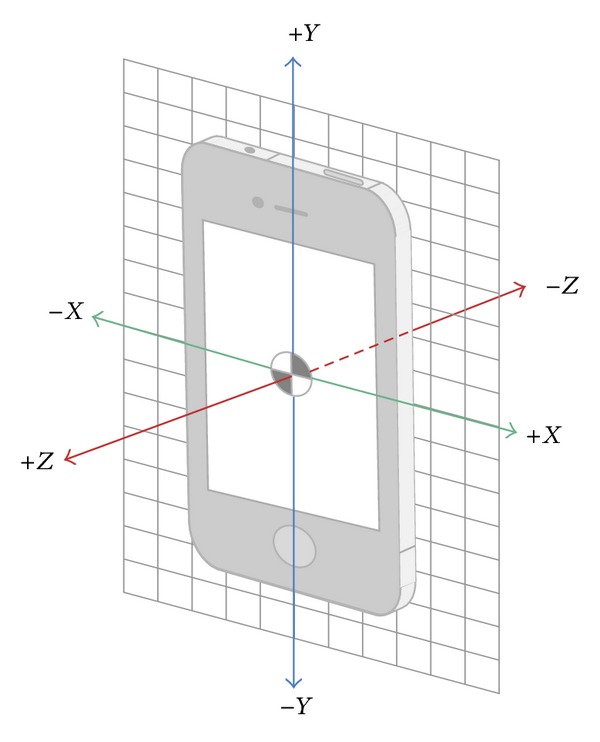
The accelerometer measures velocity along the *x*, *y*, and *z* axes.

**Figure 2:**
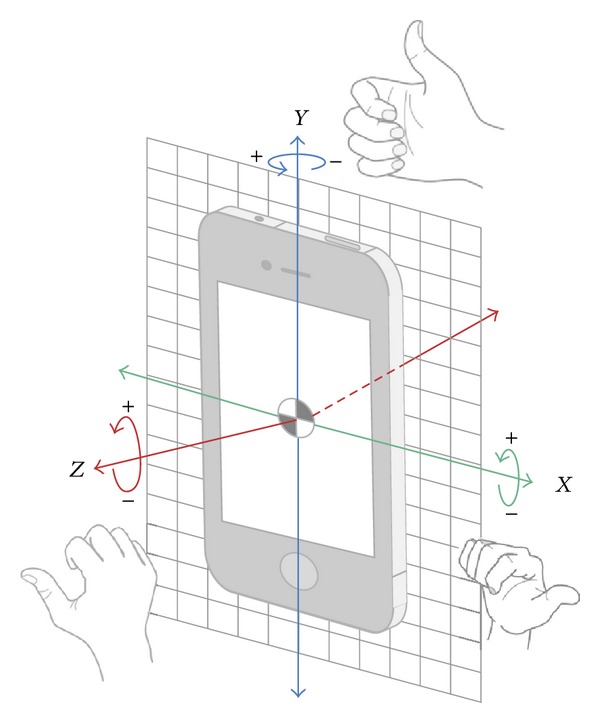
The gyroscope measures rotation around the *x*, *y*, and *z* axes.

Computerized automatic analysis was carried out to filter the inertial sensor data. This analysis, which was designed to systematically obtain kinematic data for further statistical analysis, was performed using basic software package R. Automatic analysis was guided in order to obtain kinematic information from the accelerometer and gyroscope independently for each subject in the turning transitions of the ETGUG test. The following was obtained from accelerometer: maximum peak, minimum peak, mean, and SDs of accelerations in the three axes of movements (*x*, *y*, and *z*). Also obtained were the maximum peak, minimum peak, mean, and SDs of the resultant vector (RV) accelerations (RV = √*x*2 + *y*2 + *z*2). The following was obtained from the gyroscope: maximum peak, minimum peak, mean and SDs of rotation motions in the three axis of movements (*x*, *y* and *z*). Finally, the following was obtained: maximum peak, minimum peak, mean, and SDs of the angular velocity in the three axes of movements (*x*, *y*, and *z*). The sign in the value measurements in accelerometer velocity along the *x*, *y*, and *z* axes is shown in Figure 1. The sign in the value measurements in the gyroscope rotation around the *x*, *y*, and *z* axes is shown in Figure 2. According to the information from Figure 2, if a subject performs a rotation to left during the test, the gyroscope records negative values in the *y* axes. In this study, all subjects performed the shift to the left (see Figure 2).

### 2.6. Statistical Analysis

Analysis was performed with SPSS version 15 for Windows, while data collection used inferential analysis between variables by type and normal. Mann-Whitney nonparametric tests were used, as determined by the normality of distribution variables. The statistical significance level was set at *P* < 0.05.

## 3. Results

With regard to the mean accelerometry values, Table 2 summarizes the acceleration-based measurements of the turning transitions in the ETGUG test in the two groups. Stopwatch-based ETGUG duration showed higher duration for the frail patients compared to the fit control group, as expected. The best finding in the *x*-axis was the following: the difference between groups for the minimum acceleration was 3.72 m/s^2^ (*P* < 0.01). The *y*-axis shows differences (*P* < 0.001) for maximum acceleration, 5.48 m/s^2^ between groups; the minimum acceleration was 7.44 m/s^2^ between groups. For the *z*-axis, the differences found (*P* < 0.001) were in minimum acceleration, 5.39 m/s^2^ between groups. Finally, the differences found between groups for the resultant vector values for the three accelerations show (*P* < 0.01) in the maximum, acceleration was 8.13 m/s^2^; in the minimum, acceleration was 0.78 m/s^2^; and in the resultant vector mean, acceleration was 3.08 m/s.

With regard to the mean gyroscope values, Table 3 summarizes the gyroscope-based measurements of the turning transitions in the ETGUG test in the two groups. The difference between groups for the mean maximum peak value for Yaw movement angular velocity was 86.48°/s (*P* < 0.05) (see Table 3). The difference between groups for minimum peak in angular velocity in this axis was 28.33°/s (*P* < 0.01) (see Table 3). Finally, with regard to the roll movement, the difference between groups was in the maximum angular velocity and peak was 109.04°/s (*P* < 0.01). In the minimum rotation, mean was 119.27° (*P* < 0.05). In the minimum angular velocity, peak was 19.49°/s (*P* < 0.05).

## 4. Discussion

The present study has described and examined the identification, analysis, and differentiation in the performance of kinematic variables using the inertial sensor in the *iPhone4* during the turning transitions of the ETGUG test in healthy and frail elderly persons. Significant differences were found between the groups of elderly persons in the accelerometry and angular displacement variables obtained in the kinematic readings of the trunk during the turning transitions of the ETGUG test.

The results obtained in this study show a series of deficits in the frail elderly population group. The statistically significant differences found between the groups were in the data obtained from the gyroscope and the accelerometer. From the results obtained, significant differences were obtained in the *y*-axis (Yaw movement), the *z*-axis (Pitch movement), and the *x*-axis (Roll movement).

As far as we are aware, this is the first study which has used *iPhone4* technology to analyse and study the kinematics of healthy and frail persons aged over 65 years during the turning transitions of the ETGUG test. Three recent studies [14, 25, 26] have instrumented the Timed Get Up and Go test, differentiating and analysing the kinematic data in each of the five subphases of the test between two groups of elderly persons. However, unlike the present study, they did not use *iPhone4* technology to collect kinematic variables. Their goal was to differentiate movement patterns for elderly persons with Parkinson's disease, carrying out the tests over a distance of seven meters.

It should be noted that frailty is defined as a clinical syndrome in which three or more of the following criteria should be present: unintentional weight loss, self-referred exhaustion, muscular weakness, low walking speed, and low physical activity levels [1]. Generically, the gyroscope and accelerometry data obtained for the turning transitions were similar to other studies with other types of study group. In this study, the frail elderly showed low magnitudes in the kinematic values with low variability (very small standard deviations) compared to the controls, the same as the subjects affected by Parkinson's disease [16, 25, 26], the elderly with a high risk of falls [2] and the frail elderly in a previous study [13].

Another recent study which has worked on the instrumentalization of the Timed Get Up and Go [2] test systematically evaluated the accelerometry values in elderly persons with a high risk of falls during the traditional three-meter test, focusing solely on transitions in Sit to Stand and Stand to Sit. Like the present study, this study found numerous variables from acceleration which showed differences between groups. However, in this study both the variables and the methodology, amongst other aspects, were different. Moreover, the measurement units were not coincident, and this study was based on the acceleration increased amplitude and the acceleration slope.

From a clinical perspective, the present study demonstrates that these new accelerometry parameters play an important role in differentiating between subjects with different functional states. These results provide new knowledge, extending existing knowledge of the isolated study of other transitions in frail and physically active elderly persons [12, 13, 27].

With regard to analysis of the data obtained in the present study, the differences between the frail and the physically active elderly show a series of deficits in the group of frail persons in the turning transitions. It is notable that the most significant differences in the phase were described in the results section. Moreover, as can be seen, the standard deviation in values for the frail subjects was always lower than for the physically active subjects. A previous study which analysed the behaviour of kinematic variables during turning in persons suffering from Parkinson's disease [9] did not find statistically significant differences between the groups, except in the duration of the transition. However, the present study found statistically significant differences between groups in the aforementioned variables.

Finally, it is notable that in accelerometry, three variables (minimum acceleration in the *x*, *y*, and *z* axes) showed significant differences between the groups during the turning transitions in the ETGUG test. Other studies will be required in the future in order to analyse the predictive capability of the kinematic variables which showed statistically significant differences in the different phases of the ETGUG test between healthy and frail elderly persons. This not only will help to understand which variables are of interest and are associated with the identification of the frail elderly, but also will allow early differentiation of possible pre-frail elderly which may be of use in the sphere of prevention in clinical practice.

The results obtained open up the way for further research in the future, although this study presents a series of limitations. Firstly, men and women have different characteristics, and it would be interesting to analyse differences in the kinematic data by gender following turning exercises. A new study would be required in order to compare differences by gender. Moreover, it would be interesting to consider prospective studies to determine whether the measurements obtained from trunk acceleration can predict frailty syndrome in the elderly, possibly in combination with other measurements (risk of falls). Additional work is also needed to explore other properties of accelerometer-derived measures of the turning, including comparison with gold standard. In the meantime, the present results demonstrate the potential of using an accelerometer to measure turn performance in humans, while maintaining simplicity and requiring no additional time to acquire the data.

## 5. Conclusions

The *iPhone4* inertial sensor is able to study and analyse the kinematics of the turning transitions of the ETGUG test in frail and physically active elderly persons. The accelerometry values for the frail elderly are lower than the physically active elderly, whilst variability in the readings for the frail elderly is also lower than the control group. This suggests that the frail elderly carry out the transition in a more careful, restricted way during the turning, possibly showing their reduced ability to regulate movement when performing these transitions. Compensation mechanisms also play an important role. These results indicate that the additional, relevant information for future discriminant analysis comes mainly from the acceleration signal during the different transitions of the ETGUG test.

## Figures and Tables

**Table 1:** Characteristics of sample (*n* = 30).

	Mean		SD	
	Frail (*n* = 14)	Healthy (*n* = 16)	Frail (*n* = 14)	Healthy (*n* = 16)
Age (years)	83.71	70.25	6.37	3.32
Weight (kg)	56.21	71.03	9.64	13.11
Height (cm)	155.79	159.44	7.81	10.61
Body mass index (kg/m^2^)	23.36	27.87	3.48	3.79
Total score ETGUG (s)	53.64	15.52	24.12	2.91

Kg: kilograms; cm: centimeters; m: meters; s: seconds.

**Table 2:** Acceleration-based values from the turning transition (*n* = 30).

	Mean		SD			
	Frail (*n* = 14)	Healthy (*n* = 16)	Frail (*n* = 14)	Healthy (*n* = 16)	*U*	*P* value
*t*.stopwatch (s)	5.329	2.815	1.344	2.069	2.000	**<0.001**
*x*.acc.min (m/s^2^)	−2.053	−5.779	0.962	2.433	41.00	0.003
*y*.acc.max (m/s^2^)	2.060	7.543	0.700	2.865	26.50	**<0.001**
*y*.acc.min (m/s^2^)	−2.004	−9.448	0.945	6.937	14.00	**<0.001**
*z*.acc.min (m/s^2^)	−1.815	−7.204	1.619	2.438	35.00	**<0.001**
*z*.acc.mean (m/s^2^)	−0.264	−2.903	1.553	1.331	36.00	0.002
rv.acc.max (m/s^2^)	3.634	11.985	1.165	6.523	41.00	0.003
rv.acc.min (m/s^2^)	0.621	1.403	0.672	0.980	38.00	0.002
rv.acc.mean (m/s^2^)	1.916	4.995	0.717	1.046	45.00	0.005

*x*: *x*-axis; *y*: *y*-axis; *z*: *z*-axis; acc: acceleration; *t*: time; max: maximum; min: minimum; rv: resultant vector; *U*: *U*-Mann-Whitney.

**Table 3:** Gyroscope-based values from the turning transition (*n* = 30).

	Mean		SD			
	Frail (*n* = 14)	Healthy (*n* = 16)	Frail (*n* = 14)	Healthy (*n* = 16)	*U*	*P* value
*t*.stopwatch (s)	5.329	2.815	1.344	2.069	2.000	**<0.001**
roll.rotation.min (deg)	−172.845	−53.578	12.758	64.308	60.00	0.031
roll.rotation.max (deg)	−5.770	63.360	35.422	97.818	62.00	0.038
rate.yaw.max (deg/s)	26.332	112.810	9.271	147.913	57.00	0.022
rate.yaw.min (deg/s)	−24.486	−52.809	8.867	34.733	49.00	0.009
rate.roll.max (deg/s)	25.508	134.558	14.217	135.523	13.00	**<0.001**
rate.roll.min (deg/s)	−20.396	−39.884	8.716	27.357	58.00	0.025

Max: maximum; min: minimum; *t*: time; s: second; deg: degrees; rate: angular velocity; *U*: *U*-Mann-Whitney.
